# Improvement of Maize Productivity and N Use Efficiency in a No-Tillage Irrigated Farming System: Effect of Cropping Sequence and Fertilization Management

**DOI:** 10.3390/plants10071459

**Published:** 2021-07-16

**Authors:** Heba S. A. Salama, Ali I. Nawar, Hassan E. Khalil, Ahmed M. Shaalan

**Affiliations:** 1 Crop Science Department, Faculty of Agriculture (El-Shatby), Alexandria University, Alexandria 21545, Egypt; heba.salama@alexu.edu.eg (H.S.A.S.); dralinawar@alexu.edu.eg (A.I.N.); 2 Crop Intensification Department, Field Crop Research Institute, ARC, Giza 12411, Egypt; hekhalil@gmail.com; 3 Plant Production Department, Faculty of Desert and Environmental Agriculture, Matrouh University, Mersa Matrouh 51512, Egypt

**Keywords:** maize grain yield, legume-cereal crop sequence, organic amendments, farmyard manure, mineral nitrogen, nitrogen use efficiency

## Abstract

The sequence of the preceding crops in a no-tillage farming system, could interact with the integrated use of mineral and organic nitrogen (N) sources in a way that improves the growth and productivity of the terminal maize crop, meanwhile, enhancing its N use efficiency (NUE). In the current study, six legume-cereal crop sequences, including faba bean, soybean, Egyptian clover, wheat, and maize were evaluated along two experimental rotations that ended up by planting the terminal maize crop. In addition, the effects of applying variable mineral nitrogen (MN) rates with and without the incorporation of farmyard manure (FYM) on the productive performance of maize and its NUE were tested. The field experiments were conducted in a no-tillage irrigated farming system in Northern Egypt, a location that is characterized by its arid, Mediterranean climate. Results revealed that increasing the legume component in the evaluated crop sequences, up to 75%, resulted in improved maize ear leaf area, 1000-grain weight, and harvest index, thus, a higher final grain yield, with the inclusion of Egyptian clover was slightly better than faba bean. Comparing the crop sequences with 50% legume contribution uncovered the positive effects of soybean preceding crop on the terminal maize crop. Substituting 25% of the applied MN with FYM resulted in similar maize yields to the application of the equivalent 100% MN rates. The fertilizer treatments significantly interacted with the crop sequences in determining the maize grain yield, where the highest legume crop contribution in the crop sequence (75%) equalized the effects of the different fertilizer treatments on maize grain yield. The integrated use of FYM with MN in maize fertilization improved the NUE compared to the application of MN alone. Comparing fertilization treatments with similar MN content, with and without FYM, revealed that the difference in NUE was attributed to the additional amount of FYM. In similar conditions to the current study, it is recommended to grow faba bean two years before maize, while Egyptian clover could be grown directly preceding maize growth, with frequent inclusion of soybean in the sequence, this could be combined with the application of an average of 200 kg MN ha^−1^ in addition to FYM.

## 1. Introduction 

The agricultural systems in Egypt and other developing countries are nowadays facing an unprecedented challenge in adequately feeding their continuously growing populations. Due to the extreme aridity of Egypt’s climate, agriculture is mainly dependent on irrigation [1,2], thus, concentrated along the narrow Nile Valley and Delta region. It is, therefore, crucial to plan the use and management of the limited arable land and restricted agricultural inputs to achieve the maximum benefit from the farming practice.

Among the efficient conventions and techniques utilized to achieve this goal is manipulating the order and frequency in which crops are grown, known as crop rotation [3]. Crop rotation maximizes productivity and land-use efficiency by growing different crop species in a particular sequence. Diversification of crop species through varied crop sequences and associations is an important principle of conservation agriculture that is widely advised to make the cropping systems more productive [4] and adaptive to climate stresses [5]. A well-designed crop rotation, coupled with minimum soil disturbance (no-tillage), enhances the productivity of the component crops through crop residue retention [6]. In addition, it contributes to weed and pest control [7] and improves the soil’s physical and nutritional qualities by reducing soil erosion, improving soil water holding capacity [8], and fortifying the soil organic matter [9,10]. The presence of both cereals and legumes is very common in a crop rotation that is designed to enhance productivity and increase the economic value of the farming system [11,12].

Maize (*Zea mays* L.) is a principal annual cereal crop that occurs as a main component in the crop rotations in Egypt and other Mediterranean countries. Maize, a member of the Poaceae family, is the third-largest cereal crop produced worldwide after wheat and rice with a total production of 1148 million tons from a total harvested area of 197 million hectares in 2019 [13]. In Egypt, maize plays an important role in the rural economy and livelihood [14]. It occupies a considerable area of the cultivated arable land, around 995 thousand hectares are annually devoted to maize cultivation in the summer, producing a total amount of 7.50 million tons [13]. Despite the continuous increase in the area devoted to maize production, a plateauing in the yielding capacity is observable over the last 10 years. In 2019, 7.49 t ha^−1^ were produced, against 7.82 t ha^−1^ in 2009 [13]. Thus, maize production practices are moving towards a strategy of maximizing maize yield by producing more crop on the same area of land to meet the increasing demands.

Crop rotation is identified as one of the most important factors affecting maize yield [15]. The significant influence of the preceding crops in determining maize growth and productivity is well documented in many parts of the world [11,16,17]. However, few studies have been conducted on the effect of crop rotation on maize yield under arid irrigated conditions [18]. In addition, the spectrum of crops chosen for designing a particular crop rotation is to a great extent variable among different sites [3]. Such management decision should account for the interaction between several input factors and the surrounding environment, and is thus, site-specific and should be tested in the site where it will be applied in [2]. Therefore, there seems to be a serious knowledge gap concerning the type and frequency of the preceding crops that can create the most suitable environment for a succeeding maize crop grown under irrigated conditions in the Mediterranean region.

Among the plant major nutrients, nitrogen (N) is the most important element required for improving maize growth and grain development [19,20]. It is evident that efficient N management is crucial for achieving high grain yields and for enhancing N use efficiency in maize plantations [16,21]. Previous investigations have reported the increase in maize yield and yield components in response to elevated levels of N fertilizer [20,21,22]. However, the long-term application of high mineral nitrogen (MN) levels is usually associated with several drawbacks to the plant [23,24], soil [25], and environment [26,27]. These problems may be partially avoided by the application of the different kinds of organic amendments, where significant improvements to the physical, chemical, and nutritional properties of the soil have been reported [25,28]; hence, enhancing crop productivity [29,30,31] and circumventing negative environmental implications [32]. However, the application of only organic fertilizers would not be sufficient to uplift the crop’s yield due to their low and/or unbalanced nutrient content [32,33], yet the partial substitution of mineral fertilizers with locally available organic fertilizers is a practice that is receiving increased attention in crop production [25,34]. Animal manure scored several advantages when integrated with mineral fertilizers in maize cultivation systems. It increased N uptake and crop yield [35] and also improved water use efficiency [36,37]. When partially substituting MN, the optimum combination rate of organic manure with MN is dependent on several factors, amongst is the preceding vegetation, especially under no-tillage practices, which restore the residues of the preceding crops into the soil [38]. The incorporation of legume break crops in the cropping sequence can modify the soil environment and change the needs of the maize crop to external N sources [39].

In the current study, it was hypothesized that manipulating the sequence of the preceding crops in rotation with maize would interact with the use of mineral and organic N sources in a way that uplifts the growth and productivity of the terminal maize crop, while positively altering the N use efficiency. The experiment was, therefore, set up to test, in particular, the effect of six legume-cereal crop sequences and the use of variable MN rates with and without the incorporation of farmyard manure (FYM) on the growth parameters and grain yield of maize grown under a no-tillage farming system in Northern Egypt.

## 2. Results

Mean squares presented in Table 1 and Table 2 revealed that the investigated crop sequences showed a highly significant (*p* < 0.01) influence on all the studied parameters except plant height, ear height, and leaf area index (*p* > 0.05) in the two growing seasons. Moreover, all the investigated parameters were highly significantly (*p* < 0.01) affected by the fertilization treatments except, plant height and leaf area index that were significantly (*p* < 0.05) affected, and ear height that was non-significantly affected in both seasons. Meanwhile, the leaf area index (*p* < 0.05), ear grain weight, and grain yield (*p* < 0.01) were significantly variable as affected by the two-way interaction between the crop sequence and fertilization treatment. The main effects of the two studied factors will be presented and discussed only when their interaction is not significant.

### 2.1. Crop Sequence-Related Effects

**Figure 1:**
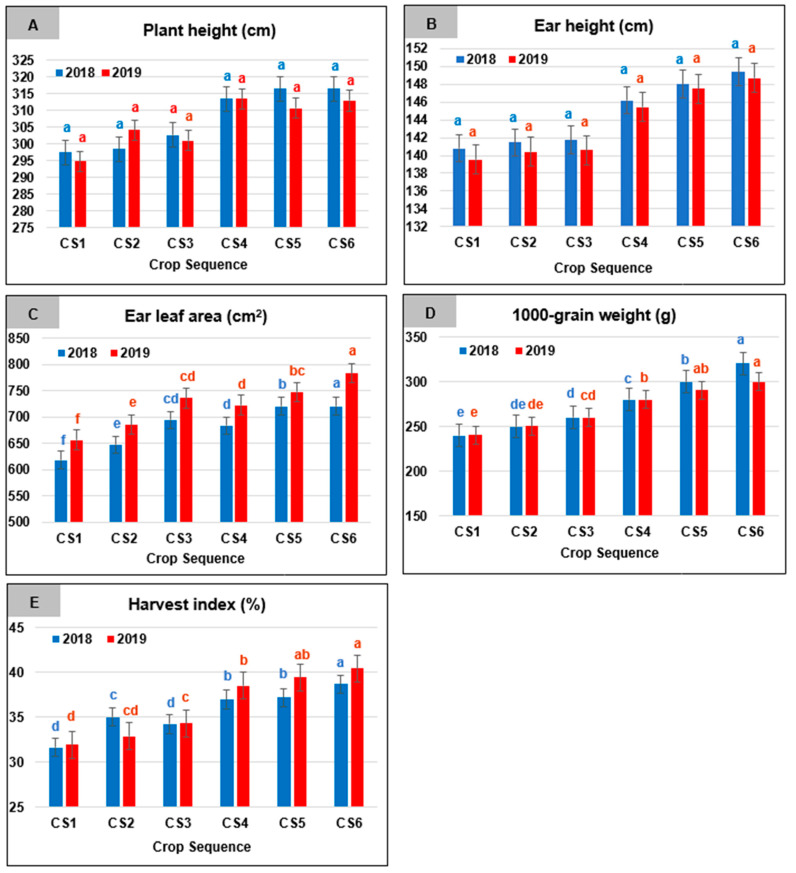
Means of plant height (**A**), ear height (**B**), ear leaf area (**C**), 1000-grain weight (**D**), and harvest index (**E**) for maize as affected by the crop sequence (CS) during 2018 and 2019 growing seasons. Means followed by a different small letter(s) within the same studied parameter for each growing season are significantly different according to the LSD test at the 0.05 level of probability.

Means of the studied parameters as affected by the crop sequences are illustrated in Figure 1. Data showed non-significant variations in plant (Figure 1A) and ear (Figure 1B) heights among the six evaluated crop sequences in both seasons. Plant height (cm) values ranged from 316.60 (CS5 and CS6) to 297.60 (CS1) and from 313.60 (CS4) to 294.90 (CS1) for 2018 and 2019, respectively, while, for the two respective seasons, values of ear height (cm) ranged from 149.39 (CS6), to 140.81 (CS1), and from 148.68 (CS6) to 139.55 (CS1).

The highest significant ear leaf area (Figure 1C) was reported for CS6, amounting to 756.60 and 782.94 cm^2^, for 2018 and 2019, respectively, against only 618.20 and 657.00 cm^2^ for CS1 during the two respective seasons. Noticeably, CS2 was also characterized by low ear leaf area compared to the other tested crop sequences.

There were significant variations in 1000-grain weight among all crop sequences (Figure 1D). Data revealed that CS6, during both seasons produced the highest significant values for that trait among all investigated crop sequences. On the other hand, CS1 was significantly inferior during both years. The highest value for 1000-grain weight was recorded for CS6 and amounted to 320.82 and 300.31 g, for the two respective seasons. These values presented an increase of around 33.47, and 24.85%, over the lowest values reported for CS1, during 2018 and 2019, respectively.

Regarding the HI, means (Figure 1E) showed that the highest significant values were obtained for CS6 (38.68%) during 2018 and for CS5 (39.40%) and CS6 (40.43%) during 2019. On the contrary, the lowest significant HI was a characteristic of CS1 (31.65%) and CS3 (34.24%) during 2018 and CS1 (31.96%) and CS2 (32.91%) during 2019.

### 2.2. Fertilization Treatment-Related Effects

**Figure 2:**
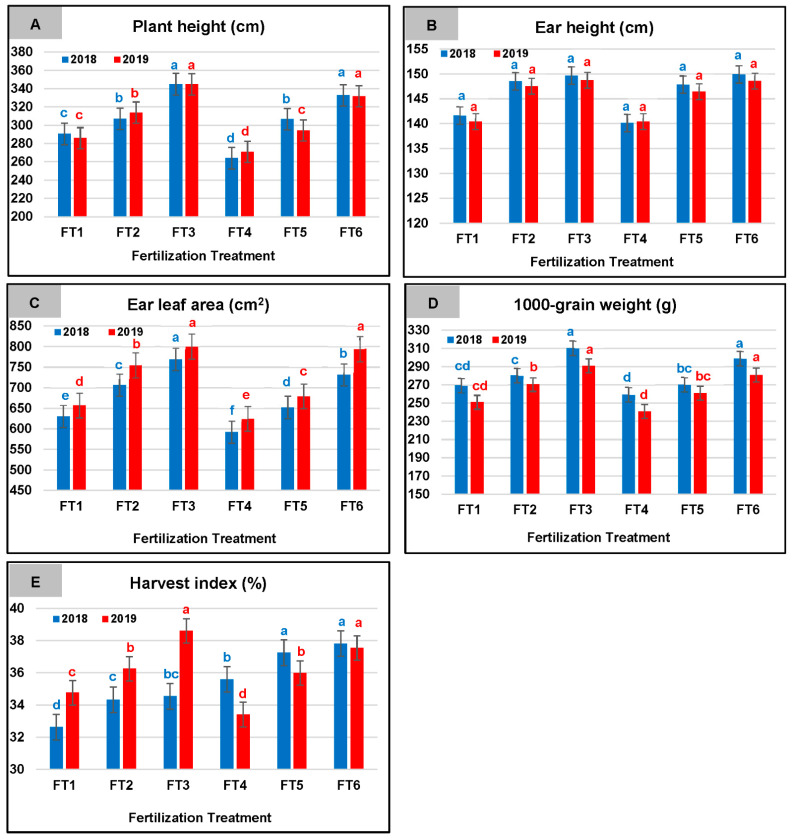
Means of plant height (**A**), ear height (**B**), ear leaf area (**C**), 1000-grain weight (**D**), and harvest index (**E**) for maize as affected by the fertilization treatment (FT) during 2018 and 2019 growing seasons. Means followed by a different small letter(s) within the same studied parameter for each growing season are significantly different according to the LSD test at 0.05 level of probability.

It was clear from the means presented in Figure 2A, that the tallest significant maize plants were observed with the application of the highest MN rate (FT3), and reached 344.80, and 344.70 cm, for 2018 and 2019, respectively. Decreasing the MN rate by 25% accompanied by the addition of FYM (FT6) resulted in significantly similar plant height to that achieved with FT3. On the other hand, the shortest significant maize plants were achieved with the application of the lowest MN rate with the application of FYM (F4) and reached 264.00, and 270.78 for 2018, and 2019, respectively. Nonetheless, intermediate and similar plant height values were reported for the application of the intermediate MN rate (FT2), and for reducing it by 25% in addition to FYM (FT5).

Ear height was non-significantly variable among the tested fertilization treatments and ranged from 140.10 cm (FT4) to 149.90 cm (FT6), during 2018, and from 140.40 cm (FT4) to 148.72 cm (FT3) during 2019 (Figure 2B). The highest significant ear leaf area was achieved with the application of the highest MN rate (FT3) during the respective seasons, reaching 769.30 and 800.44 cm^2^, in addition to FT6 during the second season (794.26 cm^2^). Although FT1 and FT2 resulted in intermediate ear leaf area, their reduction by 25% in addition to FMY (FT4 and FT5) resulted in significantly smaller ear leaf area during both seasons, with the lowest significant values reported for FT4, amounting to 592.00 and 624.52 cm^2^ for 2018 and 2019, respectively. These values represented a 23.05 and 21.98% decrease in the ear leaf area than the highest significant values reported for FT3, during the two respective seasons (Figure 2C).

Data in Figure 2D showed that the substitution of 25% of the applied MN fertilizer with FYM, resulted in significantly similar 1000-grain weights to the application of the full rates of MN without FYM, where FT1, was significantly at par with FT4, same for FT2 with FT5, and FT3 with FT6 for the two growing seasons. In general, the lowest significant 1000-grain weight was achieved with the application of FT1, and FT4, while FT3 and FT6 resulted in the highest significant 1000-grain weight for the two years. The difference between the highest and lowest values for 1000-grain weight reached 59.76 and 50.07 g for 2018 and 2019, respectively.

Harvest index (%) of maize varied significantly among all the tested fertilization treatments during both seasons (Figure 2E). The highest significant values were reported for FT5 and FT6 during 2018, and for FT3 and FT6 during 2019. On the other hand, the lowest MN rate alone (FT1) resulted in the lowest significant HI (32.63%) during 2018, while during 2019, the lowest significant HI accompanied the application of the lowest MN rate with FYM (FT4) and reached 33.42%. 

### 2.3. Interaction-Related Effects

Leaf area index (LAI) means presented in Table 3 revealed that CS4, CS5, and CS6 were superior to the other crop sequences across all tested fertilization treatments. Comparing the fertilization treatments under each crop sequence showed more pronounced variations in the direction and magnitude. Observably, under CS3 and CS5 differences among the six tested fertilization treatments diminished and they all resulted in non-significantly different LAI. Meanwhile, FT2 and FT5 were among the superior fertilization treatments for all crop sequences except CS6. Noticeably, under CS6, only FT3 and FT6 were significantly superior over the other fertilization treatments. Nonetheless, except for CS3 and CS5, FT4 resulted in the lowest significant LAI across the other four evaluated crop sequences during both seasons.

A similar trend was observed for the ear grain weight, as affected by the interaction between the crop sequence and fertilization treatment (Table 4). While CS5 and CS6 resulted in the production of the highest significant ear grain weight across all fertilization treatments during both seasons, in addition to CS4 during 2019, the lowest significant values for the trait were produced under CS1 across all fertilization treatments during both seasons, accompanied with CS2 under FT3 and FT5 during 2018, and with CS2 under FT2, FT3, FT5, and FT6 during 2019. In addition, during both seasons, FT3 and FT6 produced the highest significant ear grain weight across all crop sequences, followed by FT2 and FT5 for most of the evaluated crop sequences, while FT4 was significantly inferior to the other fertilization treatments and produced the lowest significant ear grain weight across all crop sequences.

Means of maize grain yield as affected by the interaction between the evaluated crop sequences and fertilization treatments are presented in Table 5. Data of the 2018 growing season, revealed that under the fertilization treatments with the lowest MN rate (FT1 and FT4), crop sequences CS2 to CS6 were non-significantly different and produced the highest significant grain yield, compared to CS1 which was statistically inferior. Meanwhile, when the MN rate in the fertilization treatments was increased, with or without FYM (FT2, FT3, FT5, and FT6), the crop sequences CS4 to CS6 were significantly superior to the others. On the other hand, during 2019, CS5 and CS6 were superior across all fertilization treatments, in addition to CS4 for FT1, FT3, FT5, and FT6. Despite the consistent direction of variation among the crop sequences across the fertilization treatments in both years, it was observed that a lower magnitude of variation (difference between highest and lowest values) was reported for FT1 and FT4 compared to the other treatments which might have contributed to the significant interaction. Oppositely, regarding the variations among the fertilization treatments, inconsistent direction, as well as magnitude of variation was detected. Noticeably, little variations were detected in 2018, only with CS6, FT4 was significantly inferior to the other fertilization treatments with 6.82 t ha^−1^. However, more pronounced variations among fertilization treatments were observed in 2019. It was observed that the lowest significant grain yields under CS1, CS2, and CS3 accompanied the application of FT4, amounting to 5.71, 5.91, and 6.02 t ha^−1^, for the three respective crop sequences. Nonetheless, at CS4, CS5, and CS6, FT3, FT5, and FT6 produced the highest significant grain yield.

### 2.4. Nitrogen Use Efficiency

**Figure 3:**
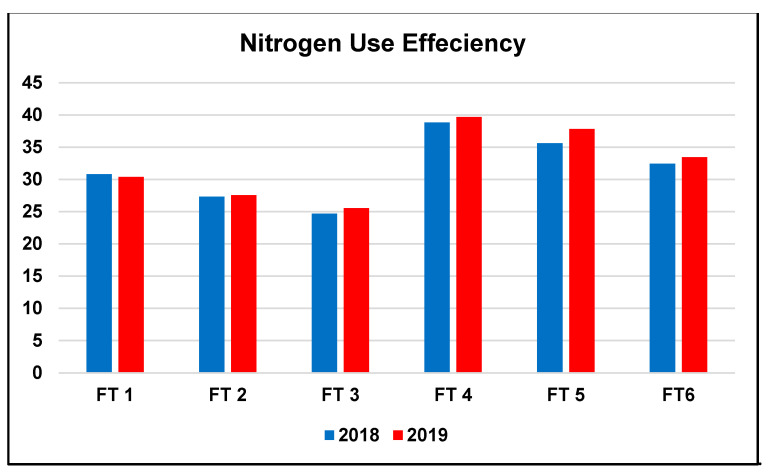
Nitrogen use efficiency of the fertilization treatments (FT) during the 2018 and 2019 growing seasons.

Data of the NUE for the evaluated fertilizer treatments during 2018 and 2019 are illustrated in Figure 3. Noticeably, comparing the three pure MN fertilization treatments revealed a decrease in the NUE with increasing the MN rate. NUE reached 30.59, 27.44, and 25.11%, on average for 2018 and 2019, for FT1, FT2, and FT3, respectively. Variations were also detected among the other three fertilization treatments with an integrated application of MN and FYM, where again, a progressive decrease in NUE accompanied increasing the MN rate in the treatment, reaching 39.26, 36.71, and 32.94, for FT4, FT5, and FT6, respectively, in average for both seasons. Nonetheless, comparing each FT composed of only MN to the adjacent treatment with 25% less MN + FYM revealed that substituting 25% of the applied MN by organic nitrogen, resulting from the addition of FYM, improved the NUE of the fertilization treatment. The difference between FT1 and FT4 was 8.67% on average for both seasons. Meanwhile, the highest percentage improvement in NUE was observed in the case of FT5 compared to FT2 and reached 9.27%. Similarly, NUE accompanying FT6 was 7.83% higher than that with FT3.

## 3. Discussion 

### 3.1. Influence of the Species and Order of the Preceding Crops in the Crop Sequence

The effect of variable legume-cereal crop sequences on the yielding potential of the terminal maize crop in a rotation is well documented by several researchers [11,16,17], yet it is greatly dependent on the species and order of the break crops in the sequence. Legume crops are recognized with three main functions in the cropping sequences: (1) provide protein-rich food and feed, (2) supply N to the system through the symbiotic N_2_ fixation, and (3) maintain the essential diversification to the cropping system (as break crops) that would uplift the productivity of the system, compared to cereal-cereal rotations [40]. Based on these functions, the three legume crop components (Faba bean, Egyptian clover, and soybean) included in the current study were chosen. The three legume crops represent important pillars in the crop rotations in Egypt and worldwide. While faba bean is a typical grain legume that is mainly grown as a protein source for food and feed [40], soybean is a prominent oil crop [41], and Egyptian clover is the main forage legume in Egypt and the Mediterranean region [42].

In the current study, increasing the legume component in the evaluated crop sequences, up to 75% (CS5 and CS6), resulted in improved maize growth parameters and, thus, higher final grain yield. This is most probably attributed to improving the soil environment, through the decomposition of the legume crop residues [43]. The ability of the legume crops to fix atmospheric N_2_ through their symbiotic association with the rhizobium bacteria allows them to play a vital role in the crop sequence. Important nutrients (like N) could be released through the mineralization of the preceding legume crop residues, thus, creating favorable conditions for the growth and activity of the useful soil microorganisms [44], and balancing their enzymatic activities [12]. In addition, legume inclusion in the cropping sequence significantly increases soil organic carbon (SOC) storage, thus soil quality [45,46]. Furthermore, some legume species are known for their ability to mobilize phosphorous from less labile phosphorous forms, thus making it more available for the succeeding cereals [47]. Meanwhile, distinguished variations were reported, in the current study, among the incorporated legume species. The incorporation of Egyptian clover in the crop sequence (CS6) showed slightly better results on the terminal maize crop, compared to the inclusion of faba bean (CS5).

Among the fertility-building plants, clovers are known for their distinguished positive impact on the succeeding cereal crops. This was reported for red, white, crimson, and Egyptian clovers [48,49]. Forage legumes, like clovers, are known to have a faster decomposition rate than grain legumes, like faba bean, thus they return N into the soil, upon mineralization, at a faster rate which favors the growth of the succeeding cereal crop [50]. This is directly linked to the low C:N ratio (around 13.7:1) characterizing the clovers among other legume crops. The lower the C:N ratio of the crop, the faster is its decomposition in the soil leading to higher N mineralization and transfer to the succeeding crops [49].

The reported benefits of including faba bean in crop sequences with cereals, are not limited to N_2_ fixation, but also include reducing the incidence of grassy weeds, pests, and diseases [40,51]. Despite those benefits, faba bean as a preceding crop usually requires a longer time than other legumes to show a significant impact on succeeding maize productivity. According to [52], the positive influence of the preceding faba bean was clear on the third succeeding maize crop. Similarly, [53] reported a significant yield increase in the second cereal following faba bean. Nonetheless, the vigorous taproot of faba bean, compared to other legume crops, could reduce the soil strength for the directly succeeding crop [51]. Applying these assumptions to our results suggests that the first sown faba bean in all sequences was able to induce a more pronounced influence on the terminal maize crop than the late sown faba bean in CS5. This also explains that comparing the crop sequences with 50% legume contribution revealed that, CS2 (with faba bean grown directly before maize) was inferior to CS4 (with soybean included in the sequence). In fact, the sequence where soybean (CS4) was utilized was superior to the other two sequences displaying the same 50% legume component percentage (CS2 and CS3). This adds value to the inclusion of soybean in the crop sequences and suggests that the positive effects associated with CS5 were most probably because of the inclusion of soybean rather than the late sown faba bean. [11], highlighted the positive impact of the preceding soybean crop in increasing the yield of succeeding maize crop, through increasing the N uptake in maize, to the extent that N became a non-limiting factor in maize productivity. Similar results were reported by [10], who documented the significant effect of soybean, occupying different sequential orders in rotation with cereals, on the performance of the succeeding maize crop. They attributed this impact to the higher content of organic carbon associated with the inclusion of soybean in the crop sequence preceding maize [38].

The choice of the break crops to be included in a particular crop sequence should be juxtaposed against the expected profitability from the whole rotation [54]. Thus, from the economic point of view, including a multi-cut forage legume like Egyptian clover, along with a main oil crop like soybean, enclosed in between a grain legume (faba bean) and a cereal crop (Maize) like in the case of CS6, provides the degree of diversification that would ensure the maximum profitability from the crop rotation, especially to smallholder farmers in the developing countries. A special emphasis is also made on CS4, where soybean is included, while Egyptian clover is replaced by wheat, which is the most important cereal crop in Egypt and the world, putting into consideration the importance of maneuvering the disadvantages associated with the cultivation of two successive cereal crops.

### 3.2. Influence of the Integrated Use of Farmyard Manure with Variable Mineral Nitrogen Rates

The ideal N management improves soil fertility, optimizes crop productivity [55], and increases NUE while reducing the potential N loss and its negative environmental implications [21,56]. In the current study, the highest MN rate (FT3) was probably sufficient to reduce interspecific competition for soil N, thus resulting in higher biomass accumulation and partitioning in maize [57]. This was denoted by the higher ear leaf area, 1000-grain weight and HI achieved with the highest MN rate, which was reflected on higher grain yield. Nonetheless, high N rates caused an increase in the LAI that was probably enough to support efficient light interception [58] and, thus, improve photosynthesis and grain setting.

Even though the highest MN rate resulted in the highest maize growth attributes and grain yield, this rate is no more recommended as it is associated with the production of the tallest significant maize plants, making the crop more prone to lodging [20]. Nonetheless, the long-term application of MN causes several soil problems, like reducing SOC and increasing soil acidity [59,60], as well as the adverse environmental effects usually accompanying the application of the high MN rates. In addition, the continuously increasing prices of MN fertilizers are putting on an additional financial burden on the smallholder farmers in the low-input agricultural systems [61].

Applying soil organic amendments is nowadays thought of as one of the efficient solutions to the above-mentioned obstacles. However, controversial results were reported in previous studies concerning the effect of incorporation of organic amendments in maize fertilization scheme on grain yield and yield components. While, [62], reported higher yields associated with MN than organic fertilizers application, [63], and [25], reported greater maize grain yield following the combined application of MN and organic amendments. [64] observed an increase in the yield of wheat, maize, and rice following the application of chemical plus organic fertilizers; they reported an average increase in yield of 29% and 8%, compared to the application of pure organic, and pure chemical fertilizers, respectively. In the current study, substituting 25% of the applied MN rate with FYM resulted in similar maize growth attributes and grain yield to the application of the full MN rates. Hence, this practice reduced the price of the fertilization treatment, meanwhile, being safer for the environment. The yield benefits associated with the integration of FYM with MN in maize fertilization might be attributed to the variations in N immobilization from the different N sources. The rapid N release from the chemical source accompanied by the slow release from the organic source secured long-term N availability and uptake to the crop [32] and resulted in improved synchrony between the nutrient’s demand and supply [65,66]. This synchrony would increase the use efficiency of both N sources resulting in better yield [63]. Nonetheless, the additional micronutrients content in the used FYM might contribute to the alleviation of other growth limiting factors, thus positively uplifting the yield of maize [67]. It was, however, clear in the present study that FYM compensated the reduction in MN only at higher MN rates (FT5 and FT6), highlighting the fact that a minimum MN rate is always required to achieve a reasonable amount of maize yield. This assumption supported the results of [68], who observed maize yield reduction as a result of the large substitution of MN with organic N. The main reason behind this is the shortage of available N, due to its slow release from the organic sources, that often does not satisfy the crop’s high N demands during the critical growth periods [69], resulting in suppressed yields. In agreement with the current study, [68], concluded that substituting 25% of the chemical N fertilizer with organic manure was sufficient to avoid yield reduction in maize. In a similar study, [20], proposed 200 kg N ha^−1^, as the appropriate MN level for improved grain filling process and lodging resistance in maize. This supports the current findings, that FT5 and FT6, containing 188 and 214 kg ha^−1^ MN, respectively, were the most appropriate MN rates, when compensated with FYM.

In the current study, the fertilizer treatment significantly interacted with the crop sequence in determining the maize grain yield, where the highest legume crop contribution in the crop sequence (75%) equalized the effects of the different fertilizer treatments on maize grain yield. This suggests that the high legume component in the crop sequence compensated for the low N content in the fertilization treatment, mostly because of the biological N_2_ fixation by legumes. It could be, thus, concluded that maize rotation with legume crops can generate enough N in the soil to meet a large part of the demands of maize, thus, reducing the required amount of N from external sources [38]. However, this effect is dependent on several factors such as the legume species [70], its biomass production [71], and the C:N ratio in its residues [72]. A larger N contribution from Egyptian clover than faba bean is, thus, expected, since all N produced from Egyptian clover will remain in the system, while in the case of faba bean large proportion of N will be exported to the pods to support seed setting. A similar explanation was provided by [73], for vetch and field pea.

Although high N rates are needed for achieving high yields, they are associated with reduced NUE [57,74,75], most probably because the crop N utilization will be constrained by several biotic and abiotic factors that will be generated as a result of the high N rates [76]. The wise fertilization management, thus, aims at achieving the best balance between the crop’s yielding potential and its NUE. Improved NUE will, consequently, decrease the application rate of N fertilizers, thus, reduce the farming expenses and protect the environment [77]. Several previous investigations reported a decrease in NUE with the combined application of organic and mineral fertilizers, they attributed this to the higher total N content of the combined fertilizer [78], or the lower rate of recovery for the N of organic origin in the first year of application leading to lower NUE, while higher residual benefits could be observed in subsequent years [63]. On the contrary to the previous results, the combination of FYM and MN, in the present study, significantly increased the NUE. Calculating the N content in those treatments, revealed that the combination of 25% reduced MN content with the FYM containing 35 kg N ha^−1^ (FT4, FT5, FT6) resulted in a lower total N content than the application of the 100% MN rates (FT1, FT2, FT3), yet a higher NUE and grain yield. Moreover, results indicated that, despite similar MN content, yet higher total N content, FT6 was superior to FT1 in improving NUE, suggesting that this difference in NUE was attributed to the additional amount of FYM in the FT6. This implies that the N from organic sources does not have a similar detrimental effect on NUE as the same amount of N from mineral sources.

## 4. Materials and Methods

### 4.1. Site Characteristics

**Figure 4:**
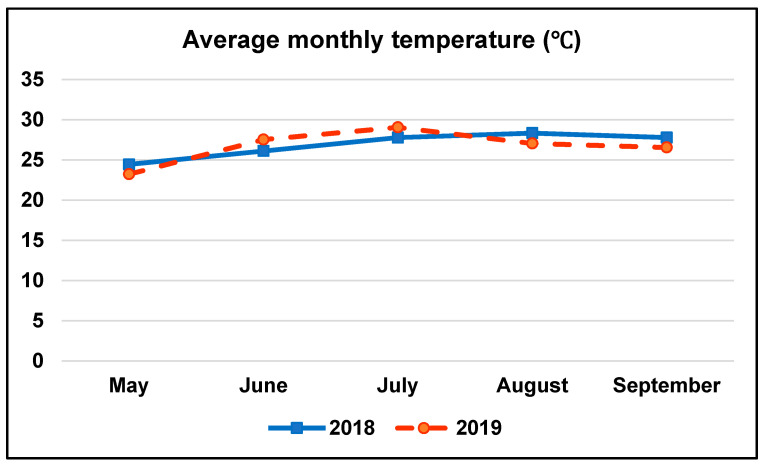
Average monthly temperature (°C) for 2018 and 2019 growing seasons.

**Figure 5:**
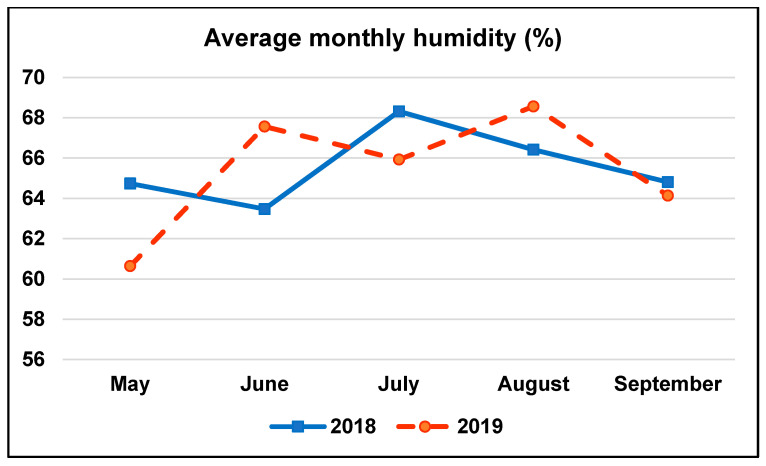
Average monthly humidity (%) for 2018 and 2019 growing seasons.

A field experiment was carried out over two periods of time; from 2016 to 2018, and from 2017 to 2019 at the Agricultural Research Station, Crop Science Department, Alexandria University, located at 31.22° N, 29.94° E. The experimental location was characterized by its Mediterranean climate, average monthly temperature (°C), and humidity (%) for the two summer seasons (2018 and 2019), where the terminal maize crop was planted, are presented in Figure 4 and Figure 5, respectively. The physical and chemical characteristics of the experimental soil are presented in Table 6.

### 4.2. Experimental Design and Treatments

A factorial layout of treatments in a three-replicated randomized complete block design (RCBD) was adopted for the field experiments, to investigate the effect of six crop sequences and six fertilization treatments on the growth and productivity of the terminal maize crop. Six crop sequences were investigated in the current study, in which three different crops were grown along the experimental periods, that ended up with growing the terminal maize crop. The different crops and cultivars included in the six investigated sequences were: Maize (Giza 168), faba bean (Giza 843), soybean (Giza 111), wheat (Gemmeza 9), Egyptian clover (Helaly). The studied crop sequences in both experimental periods are presented in Table 7.

Six fertilization treatments were investigated in the current study and included three mineral nitrogen (MN) full rates. The other three fertilization treatments included 25% less MN rates than the previous three treatments plus a fixed amount of organic farmyard manure (FYM). Thus, the investigated fertilization treatments were as follows; FT1 = 215 kg N ha^−1^, FT2 = 250 kg N ha^−1^, FT3 = 285 kg N ha^−1^ (the recommended N rate for maize growing in the region), FT4 = 161 kg N ha^−1^ + 9.6 t ha^−1^ FYM, FT5 = 188 kg N ha^−1^ + 9.6 t ha^−1^ FYM, FT6 = 214 kg N ha^−1^ + 9.6 t ha^−1^ FYM. The mineral nitrogen (MN) was applied in the form of ammonium nitrate (33.5% N). The FYM was added at a rate of 9.6 t ha^−1^, containing 3.60 and 3.21 kg ton^−1^ nitrogen and phosphorus respectively. Thus, the added amount of FYM (9.6 t ha^−1^) provided around 35 kg organic N ha^−1^. FYM was manually incorporated into the soil 3 weeks before sowing of the terminal maize crop. The used FYM had a pH value of 6.9, EC value of 4.3, and 17.6, 1.18, 1.07%, for total C, N, and P, respectively, in addition to 14.92 C/N ratio.

### 4.3. Crop Establishment and Management Practices

**Figure 6:**
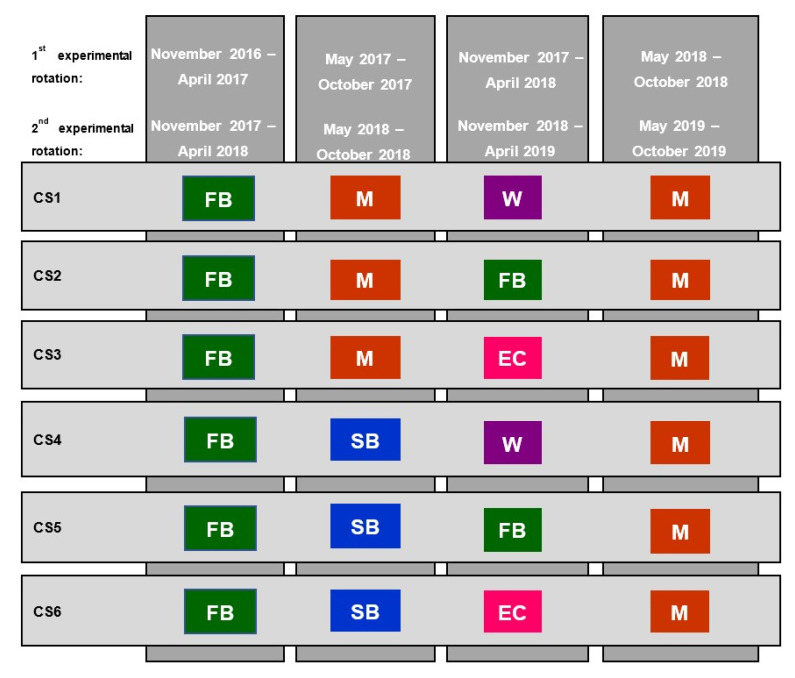
Sowing orders and dates of the component crops in the evaluated crop sequences (CS) across the two experimental rotations. M = Maize, FB = Faba bean, SB = Soybean, W = Wheat, EC = Egyptian clover.

The first experimental rotation was initiated in winter 2016/2017 and terminated in summer 2018, while the second experimental rotation started in winter 2017/2018 and ended in summer 2019. Both experimental rotations ended up by planting the terminal maize crop. A fixed layout was maintained since the start of the experiment and applied to both experimental rotations. A schematic diagram of the evaluated crop sequences in the two experimental rotations illustrating the sowing order and dates of the different crop components is given in Figure 6.

The experimental plot comprised four raised wide beds, each was 3 m long and 1.2 m wide, resulting in a total plot area of 14.4 m^2^. All crops were planted following the recommendations in the region regarding sowing method and seeding rate. Wheat was drilled on the upper side of the raised wide bed, in rows 30 cm apart, with a 144 kg ha^−1^ seeding rate. Two grains of Maize were sown in hills (30 cm apart) and thinned to one plant per hill 14 days after sowing to maintain the recommended plant density (57,600 plant ha^−1^). Egyptian clover was broadcasted with a 48 kg ha^−1^ seeding rate. Faba bean and soybean were sown in hills, that were 30 and 20 cm apart, respectively, and in rows (30 cm spaced), and thinned to two plants per hill, 14 days after sowing for both crops. The used seeding rate was 96 and 72 kg ha^−1^, for faba bean and soybean, respectively. Prior to sowing, seeds of faba bean, soybean, and Egyptian clover were inoculated with the appropriate *Rhizobium* spp. to enhance biological N_2_ fixation. The specific strains, namely; *Rhizobium leguminosarum* bv. *viciae*, *Bradyrhizobium japonicum*, and *Rhizobium trifolii* were used to inoculate faba bean, soybean, and Egyptian clover seeds, respectively. A zero-tillage strategy was adopted along with the two experimental rotations, to avoid any soil disturbance and keep the preceding crop residues in the soil. 

All component crops in the evaluated crop sequences were fertilized upon the recent recommendations of the Egyptian Ministry of Agriculture and Land Reclamation. The recommended phosphorus rate of 36 kg P_2_O_5_ ha^−1^, applied as calcium monophosphate (15.5% P_2_O_5_), was added to the soil with seedbed preparation. Meanwhile, nitrogen in the form of ammonium nitrate (33.5% N), was added as 36 kg N ha^−1^ for faba bean, Egyptian clover, and soybean, 144 kg N ha^−1^ for wheat, and 285 kg N ha^−1^ for maize, which was split into two equal applications, added with the first and second irrigations. The terminal maize crop received the recommended phosphorus rates, in addition to the tested fertilizer applications as a source of organic and mineral N. To avoid induced drought stress, surface irrigation was scheduled on 14- and 7-day intervals during the winter and summer seasons, respectively. No serious incidence of pests or diseases was observed along the experimental periods, and hand weeding was always practiced with all the crop components.

### 4.4. Maize Harvesting and Measurements

At grain full maturation and prior to harvesting of the maize terminal crop, plant height (cm), ear height (cm), ear leaf area (cm^2^), and leaf area index were determined for five randomly chosen plants from each experimental plot. At harvesting, the stalks of the maize plants on the inner two wide beds for each plot were manually cut with a sickle directly above ground level, then the total biological yield was immediately weighed in the field. After removing the ears from the plants, they were separately shelled and grain yield was weighed as kg plot^−1^, in addition, the ear grain weight (g) was calculated as an average of five random ears from each plot. The 1000-grain weight (g) was determined for three random grain samples taken from each plot. Harvest index (HI) was calculated as grain yield divided by total biological yield and expressed as a percentage.

Nitrogen use efficiency (*NUE*) was calculated after [79] as the grain yield obtained per unit of applied mineral N fertilizer as follows:

*NUE* (%) = [*G_f_*/*MN_a_*] × 100

where *G_f_* is the grain yield of the fertilized plot, and *MN_a_* is the total amount of applied mineral N at each fertilization treatment.

### 4.5. Statistical Procedures

The crop sequences, fertilizer treatments, and their interaction were tested for significance using Proc Mixed of SAS 9.4 [80] after [81]. Only replicates were considered random. The investigated parameters of maize growth and productivity (*p*) were analyzed according to the following model:

*P_ijk_* = *µ* + *R_i_* + *CS_j_* + *FT_k_* + (*CS* × *FT*)*_jk_* + *e_ijk_*

where *µ* is the overall mean, *R_i_* is the replication (*i* = 1, 2, 3), *CS_j_* is the crop sequence effect (*j* = 1, 2, 3, 4, 5, 6), *FT_k_* is the fertilization treatment effect (*k* = 1, 2, 3, 4, 5, 6), (*CS* × *FT*)*_ij_* is the effect of the interaction between the crop sequence and fertilization treatment, and *e_ijk_* is the experimental error.

Prior to the statistical analysis of the data, the harvest index was arcsine transformed and expressed as a percentage. Means were compared using the least significant difference (L.S.D) procedure, with significances declared at *p* < 0.05.

## 5. Conclusions

The present research highlighted opportunities for the improvement of maize productivity when grown in a no-tillage farming system, in an arid Mediterranean region, through manipulating the preceding crop sequence and N fertilizer input. Increasing the legume component in the evaluated crop sequences, led to higher maize grain yields, with the inclusion of Egyptian clover being slightly better than faba bean. Especial emphasis was made on the importance of including soybean in the crop sequence preceding the terminal maize crop. Substituting 25% of the applied MN with FYM resulted in similar maize yields to the application of the equivalent 100% MN rates, under high MN rates, thus, a minimum MN rate is always required to achieve a reasonable amount of maize yield. The highest legume crop contribution in the crop sequence (75%) equalized the effects of the different fertilizer treatments on maize grain yield. The integrated use of FYM with MN in maize fertilization improved the NUE compared to the application of MN alone, suggesting that the N from organic sources does not have a similar detrimental effect on NUE as the same amount of N from mineral sources. In similar conditions to the current study, it is recommended to grow faba bean two years before maize, while Egyptian clover could be grown directly preceding to maize, with a frequent inclusion of soybean in the sequence and this could be combined with the application of an average of 200 kg MN ha^−1^ in addition to FYM.

## Figures and Tables

**Table 1:** Mean squares and levels of significance of the plant height (cm), ear height (cm), ear leaf area (cm^2^), and leaf area index (LAI) as affected by crop sequence (CS), fertilization treatment (FT), and their interaction for 2018 and 2019 growing seasons.

Source of Variations	d.f	Plant Height		Ear Height		Ear Leaf Area		Leaf Area Index	
		2018	2019	2018	2019	2018	2019	2018	2019
Crop sequence (CS)	5	425.08 ^ns^	524.18 ^ns^	299.42 ^ns^	273.78 ^ns^	1210.91 **	1420.81 **	0.22 ^ns^	0.25 ^ns^
Fertilization treatment (FT)	5	950.84 *	1011.54 *	287.14 ^ns^	340.77 ^ns^	1413.12 **	1723.66 **	0.39 *	0.35 *
CS * FT	25	274.70 ^ns^	490.20 ^ns^	298.40 ^ns^	322.16 ^ns^	550.20 ^ns^	644.33 ^ns^	0.21 *	0.23 *
Error	70	392.45	420.15	250.23	310.42	420.18	510.36	0.13	0.14

d.f.: degrees of freedom, ns: Non-significant, * Significant at 0.05 level of probability, ** significant at 0.01 level of probability.

**Table 2:** Mean squares and levels of significance of the ear grain weight (g), 1000-grain weight (g), grain yield (t ha^−1^), and harvest index (%) as affected by crop sequence (CS), fertilization treatment (FT), and their interaction for 2018 and 2019 growing seasons.

Source of Variations	d.f	Ear Grain Weight		1000-Grain Weight		Grain Yield		Harvest Index	
		2018	2019	2018	2019	2018	2019	2018	2019
Crop sequence (CS)	5	31.20 **	40.18 **	56.14 **	80.08 **	10.12 **	9.44 **	255.10 **	312.33 **
Fertilization treatment (FT)	5	33.15 **	51.02 **	60.34 **	79.12 **	12.88 **	13.40 **	195.17 **	298.54 **
CS*FT	25	17.44 **	18.56 **	20.12 ^ns^	30.45 ^ns^	5.52 **	4.20 **	41.21 ^ns^	50.18 ^ns^
Error	70	9.30	9.99	18.60	23.55	0.32	0.16	33.71	45.25

d.f.: degrees of freedom, ns: Non-significant, * Significant at 0.05 level of probability, ** significant at 0.01 level of probability.

**Table 3:** Variations in LAI as affected by the interaction between the crop sequence (CS) and fertilization treatment (FT) during 2018 and 2019 growing seasons.

Crop Sequence	Fertilization Treatment					
	Growing Season 2018					
	FT1	FT2	FT3	FT4	FT5	FT6
**CS1**	3.09 cAB	3.20 cAB	3.60 cA	2.85 dB	3.11 cAB	3.40 dAB
**CS2**	3.14 cBC	3.25 cABC	3.79 cA	3.00 cdC	3.15 cBC	3.70 dAB
**CS3**	3.59 bcA	3.72 bcA	4.08 bcA	3.50 bcA	3.70 bcA	3.90 cdA
**CS4**	3.80 abB	4.10 abAB	4.57 abA	3.78 abB	4.00 abAB	4.44 abcA
**CS5**	4.11 aA	4.40 aA	4.66 abA	4.08 abA	4.37 aA	4.59 abA
**CS6**	4.30 aC	4.49 aBC	5.12 aA	4.20 aC	4.45 aBC	4.93 aAB
**L.S.D._0.05_**	**0.59**					
	**Growing Season 2019**					
	**FT1**	**FT2**	**FT3**	**FT4**	**FT5**	**FT6**
**CS1**	3.10 cB	3.33 dAB	3.77 dA	2.95 dB	3.23 cAB	3.56 cAB
**CS2**	3.18 cBC	3.23 dABC	3.80 dAB	3.10 cdC	3.20 cABC	3.82 cA
**CS3**	3.66 bcA	3.85 cdA	4.10 cdA	3.64 bcA	3.82 bcA	4.01 bcA
**CS4**	3.94 abBC	4.13 abcABC	4.64 abcA	3.80 abC	4.10 abABC	4.52 abAB
**CS5**	4.21 abA	4.53 abA	4.74 abA	4.13 abA	4.44 abA	4.65 aA
**CS6**	4.43 aBC	4.59 aBC	5.23 aA	4.33 aC	4.54 aBC	4.98 aAB
**L.S.D._0.05_**	**0.62**					

Means followed by a different small letter(s) within the same column, or different capital letter(s) within the same row, for the same growing season, are significantly different according to the LSD test at 0.05 level of probability.

**Table 4:** Variations in ear grain weight as affected by the interaction between the crop sequence (CS) and fertilization treatment (FT) during 2018 and 2019 growing seasons.

Crop Sequence	Fertilization Treatment					
	Growing Season 2018					
	FT1	FT2	FT3	FT4	FT5	FT6
**CS1**	92.30 dB	95.41 eAB	100.11 dA	80.04 dC	97.13 dAB	96.71 eAB
**CS2**	95.67 cdC	100.39 dABC	104.00 dA	83.08 cdD	101.19 cdAB	101.82 dAB
**CS3**	100.44 cC	105.21 cdABC	110.00 cA	86.66 cD	104.21 cBC	107.21 cAB
**CS4**	115.31 bC	121.84 bB	127.34 bA	100.14 bD	121.34 bB	125.11 bAB
**CS5**	120.74 aD	127.13 aBC	133.25 aA	105.00 abE	126.42 aBC	130.30 aAB
**CS6**	125.22 aC	131.08 aAB	135.68 aA	109.30 aD	130.08 aBC	133.12 aAB
**L.S.D._0.05_**	**4.93**					
	**Growing Season 2019**					
	**FT1**	**FT2**	**FT3**	**FT4**	**FT5**	**FT6**
**CS1**	93.11 dB	97.15 dAB	100.54 cA	79.76 dC	100.34 cA	96.42 cAB
**CS2**	95.89 cdC	98.78 dBC	104.14 bcA	85.53 cD	101.67 cAB	99.66 cABC
**CS3**	98.85 cC	105.66 cAB	109.21 bA	88.86 cD	103.65 cB	106.59 bAB
**CS4**	118.60 bC	125.32 bB	132.61 aA	102.74 bD	128.44 bAB	129.64 aAB
**CS5**	122.00 bB	132.67 aA	133.85 aA	108.43 aC	131.01 bA	131.30 aA
**CS6**	130.54 aB	134.59 aAB	137.62 aA	112.66 aC	137.50 aA	134.42 aAB
**L.S.D._0.05_**	**5.11**					

Means followed by a different small letter(s) within the same column, or different capital letter(s) within the same row, for the same growing season, are significantly different according to the LSD test at 0.05 level of probability.

**Table 5:** Variations in grain yield as affected by the interaction between the crop sequence (CS) and fertilization treatment (FT) during 2018 and 2019 growing seasons.

Crop Sequence	Fertilization Treatment					
	Growing Season 2018					
	FT1	FT2	FT3	FT4	FT5	FT6
**CS1**	6.03 bA*	6.04 cA	6.28 cA	5.72 cA	5.97 dA	6.30 dA
**CS2**	6.14 abA	6.28 cA	6.43 cA	5.84 bcA	6.15 cdA	6.50 cdA
**CS3**	6.33 abA	6.51 bcA	6.83 bcA	5.94 abcA	6.58 bcdA	6.77 cdA
**CS4**	7.01 aA	7.37 abA	7.46 abA	6.57 abcA	6.89 abcdA	7.30 abcA
**CS5**	7.12 aA	7.41 abA	7.52 abA	6.69 abA	7.11 abA	7.43 abA
**CS6**	7.23 aAB	7.65 aAB	7.96 aA	6.82 aB	7.80 aA	7.90 aA
**L.S.D._0.05_**	**0.92**					
	**Growing Season 2019**					
	**FT1**	**FT2**	**FT3**	**FT4**	**FT5**	**FT6**
**CS1**	5.85 cBC	6.19 cABC	6.55 bA	5.71 dC	6.33 bABC	6.43 bAB
**CS2**	6.03 cAB	6.27 cAB	6.59 bA	5.91 dB	6.39 bAB	6.38 bAB
**CS3**	6.17 bcAB	6.49 bcAB	6.75 bA	6.02 cdB	6.59 bAB	6.61 bAB
**CS4**	6.74 abB	7.11 bB	8.02 aA	6.60 bcB	7.84 aA	7.83 aA
**CS5**	7.21 aB	7.90 aA	8.29 aA	7.10 abB	8.10 aA	8.15 aA
**CS6**	7.33 aB	8.10 aA	8.35 aA	7.30 aB	8.27 aA	8.30 aA
**L.S.D._0.05_**	**0.66**					

Means followed by a different small letter(s) within the same column, or different capital letter(s) within the same row, for the same growing season, are significantly different according to the LSD test at 0.05 level of probability.

**Table 6:** Physical and chemical analyses of the experimental soil.

Soil Texture	Sand%	Silt %	Clay %	pH	EC (dS/m)	CaCO_3_ (%)	OM (%)	Available N mg/kg	Available P mg/kg	Available K mg/kg
Sandy loam	62.3	20.0	17.7	8.30	2.10	9.87	0.50	100	9.61	31.98

**Table 7:** Order of planting the different crop components in the investigated crop sequences.

Crop Sequence	Planting Order of the Different Crop Components			Terminal Crop	Contribution Percentage in Each Crop Sequence	
					Legumes	Non Legumes
CS1	FB	M	W	M	25	75
CS2	FB	M	FB	M	50	50
CS3	FB	M	EC	M	50	50
CS4	FB	SB	W	M	50	50
CS5	FB	SB	FB	M	75	25
CS6	FB	SB	EC	M	75	25

M = Maize, FB = Faba bean, SB = Soybean, W = Wheat, EC = Egyptian clover.
